# Supplementary material for: Dynamics of Gut Microbiota and Short-Chain Fatty Acids during a Cycling Grand Tour Are Related to Exercise Performance and Modulated by Dietary Intake
Source: Nutrients. 2024 Feb 27;16(5):661. doi: 10.3390/nu16050661 (PMC10934853; doi:10.3390/nu16050661)
Supplement: Supplementary file 1 [file nutrients-16-00661-s001.zip › nutrients-2844812-supplementary/Fernandez Sanjurjo et al_Supplementary Figures (v. 2).pptx]

## Slide 1
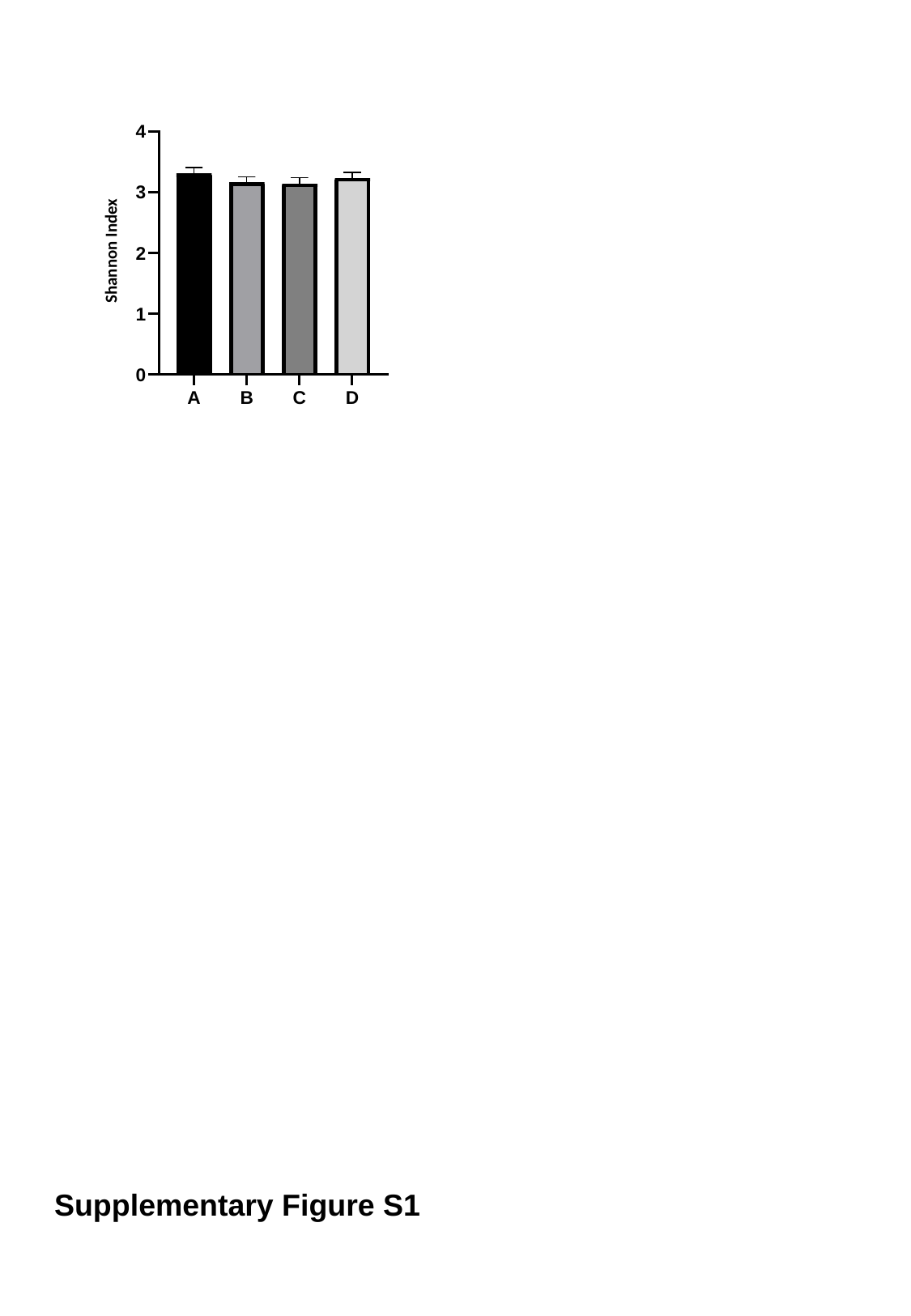

Shannon Index
Supplementary Figure S1

## Slide 2
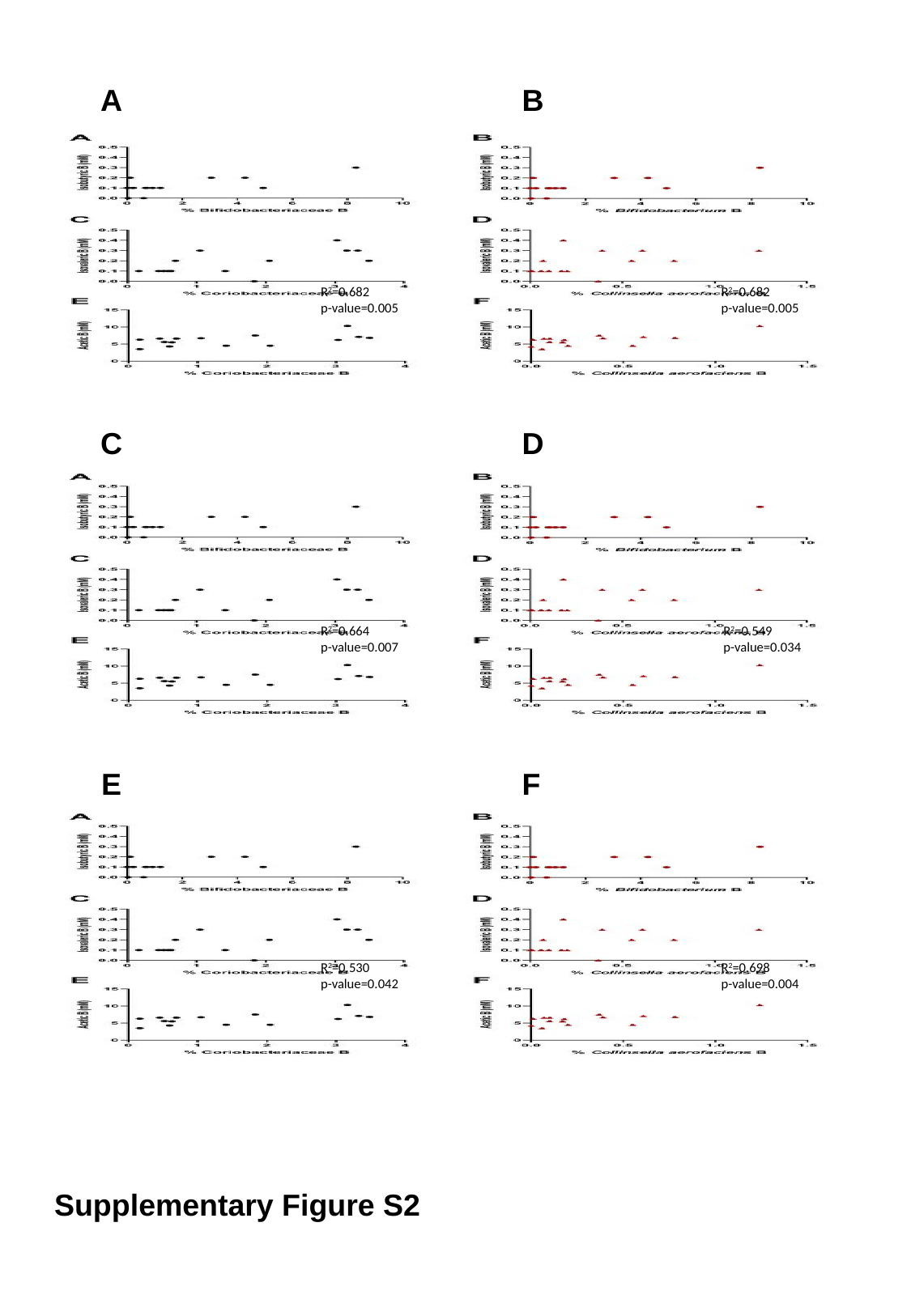

A
B
R2=0.682
p-value=0.005
R2=0.682
p-value=0.005
C
D
R2=0.549
p-value=0.034
R2=0.664
p-value=0.007
E
F
R2=0.698
p-value=0.004
R2=0.530
p-value=0.042
Supplementary Figure S2

## Slide 3
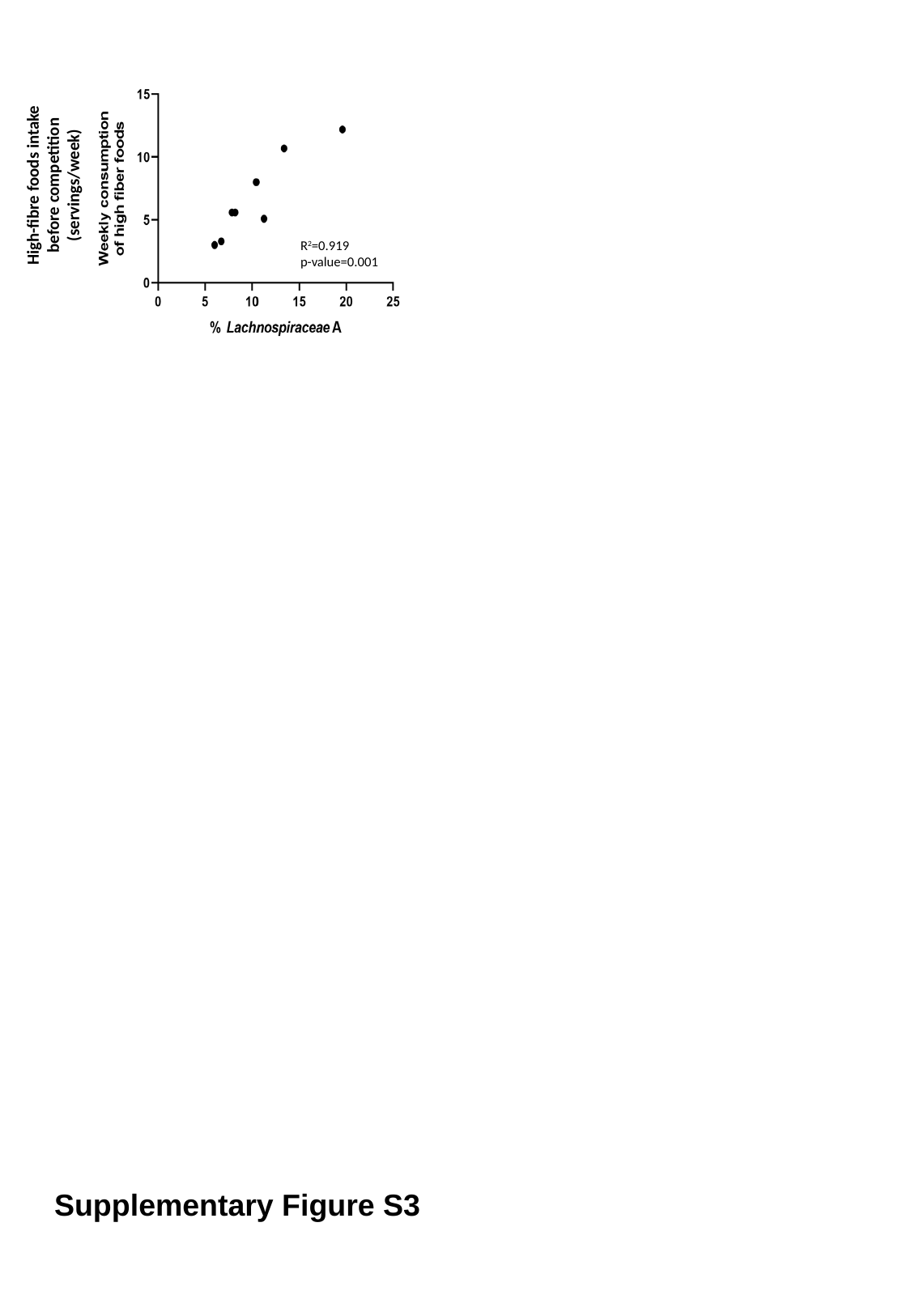

High-fibre foods intake before competition (servings/week)
R2=0.919
p-value=0.001
Supplementary Figure S3
